# Long-Term In-Service Monitoring and Performance Assessment of the Main Cables of Long-Span Suspension Bridges

**DOI:** 10.3390/s17061414

**Published:** 2017-06-16

**Authors:** Yang Deng, Yang Liu, Suren Chen

**Affiliations:** 1 School of Civil Engineering and Architecture, Changsha University of Science & Technology, Changsha 410004, China; 2 Beijing Advanced Innovation Center for Future Urban Design, Beijing University of Civil Engineering and Architecture, Beijing 100044, China; 3 Department of Civil and Environmental Engineering, Colorado State University, Fort Collins, CO 80523, USA; suren.chen@colostate.edu

**Keywords:** suspension bridge, main cable, tension force, structural health monitoring, vibrating strings transducer, safety factor

## Abstract

Despite the recent developments in structural health monitoring, there remain great challenges for accurately, conveniently, and economically assessing the in-service performance of the main cables for long-span suspension bridges. A long-term structural health monitoring technique is developed to measure the tension force with a conventional sensing technology and further provide the in-service performance assessment strategy of the main cable. The monitoring system adopts conventional vibrating strings transducers to monitor the tension forces of separate cable strands of the main cable in the anchor span. The performance evaluation of the main cable is conducted based on the collected health monitoring data: (1) the measured strand forces are used to derive the overall tension force of a main cable, which is further translated into load bearing capacity assessment using the concept of safety factor; and (2) the proposed technique can also evaluate the uniformity of tension forces from different cable strands. The assessment of uniformity of strand forces of a main cable offers critical information in terms of potential risks of partial damage and performance deterioration of the main cable. The results suggest the proposed low-cost monitoring system is an option to provide approximate estimation of tension forces of main cables for suspension bridges. With the long-term monitoring data, the proposed monitoring-based evaluation methods can further provide critical information to assess the safety and serviceability performance of main cables.

## 1. Introduction

As a type of critical infrastructure, long-span bridges are usually vital to the local traffic around the world. Structural health monitoring (SHM) techniques have often been applied to assess the conditions of these important structures [1,2,3,4]. Due to the complexity of the bridge structures, associated high cost of SHM, and performance limitations of sensors, many challenges remain in terms of how to conduct SHM and interpret the data more effectively and economically [3]. For suspension bridges, main cable is one of the most critical components and some efforts have been carried out to evaluate its performance condition through applying SHM techniques [5,6]. For instance, a model has been developed to link the horizontal tension of a main cable and measured natural frequencies [6].

Great challenges still exist in monitoring main cables’ tension forces during in-service time. It is difficult to directly measure the tension force of a main cable through the means of conventional sensing techniques due to the large cross-section size of the main cable (formed by thousands of parallel high-strength steel wires) and huge tension force values. Other studies have adopted the electromagnetic (EM) stress sensor to obtain the tension forces of a main cable based on the correlation of the relative permeability and tensile stress [7,8,9]. Due to the size limit and relatively high cost of the sensor, EM sensors were primarily used in measuring tension forces of arch bridge hangers, post-tensioned tendons [7,8] or external tendons of the bridges’ box girders [9]. In fact, the studies on long-term in-service tension monitoring of main cables of suspension bridges have been rarely reported.

Instead of directly measuring the tension force of the entire main cable, an alternative is to measure tension forces of a series of strands in the anchor span, which goes from the saddle to the anchor system in the anchorage. The measured tension forces of strands will be integrated to obtain the total force of the entire main cable. There are several approaches to measure the tension forces of individual strands. Firstly, the tension forces can be derived from the measured basic frequencies of individual cable strands [10,11]. Such a method was inspired by the vibration-based tension identification theories for stayed cables [12] and was used to control the tension forces of the strands during the construction period. Along the same line, Zhang et al. [10] established finite element models of anchor span strands of Taizhou Suspension Bridge. Then the mapping relationships between vibration frequencies and the tension forces of the strands in the anchor span were derived by finite element analysis. Wang et al. [11] further improved the formula linking the vibration frequency with tension force of the anchor span strands by considering the influence of the anchor device. Recently, vision sensors have used to identify cables’ dynamic characteristics and then the tension forces of the cables can be estimated [13,14]. This vision-based technique is a new option of vibration-based technique for cables’ tension force estimation. However, the vibration-based technique cannot carry out real-time and continuous data acquisition. Thus, the tensions of the strands obtained by this technique are discrete in time. The second technique for strand tension monitoring is Fiber Bragg Grating (FBG) force testing ring invented by Nan [15]. This kind of device can provide precise measurements but with relatively high cost. In addition, there are some emerging techniques including FBG-based smart strand [16,17] and guided ultrasonic or stress waves [18,19,20,21,22]. As of now, these techniques, however, have only been investigated in laboratories and rarely tested in bridge field monitoring practice.

Therefore, to develop a highly reliable, cost-effective, and precise system for tension monitoring of main cable is desirable. In this study, s conventional vibrating strings transducer (VST), a kind of reliable, low-cost, and technology-mature sensor for pressure measuring, is used in monitoring of a main cable. VST has already been well developed [23,24] and used with good performance in civil engineering applications such as, tension monitoring of main cable strands during construction [25], strain monitoring of building columns during construction [26] and temperature and strain monitoring of concrete bridges during construction [27]. The feasibility of adopting VST on measuring tension forces of cable strands can be partially verified by a study by Huang and Mu [25]. In their study, VSTs were used to measure and control the strands’ tension forces in the anchor span during the construction of a long-span suspension bridge. However, there is no reported study about applying the VSTs on the long-term monitoring of suspension bridges during service time.

A long-term monitoring strategy of applying VSTs on a long-span suspension bridge to collect in-service main cable force data is developed. After the VST transducers measure the forces of a series of separate cable strands, the tension of the whole main cable is further derived approximately. With the monitored data, the performance assessment of main cable forces based on the measured data is also demonstrated on a prototype bridge. One-day and seven-month monitoring data are processed and analyzed to provide some insights of main cable tension forces. The uniformity of strands’ tension forces and the main cable’s safety factor related to load bearing capacity are also evaluated.

## 2. Bridge Description and Instrumentation

### 2.1. The Nanxi Suspension Bridge (NSB)

**Figure 1:**
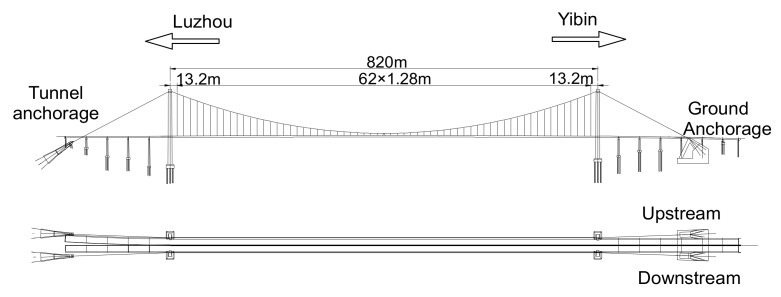
The NSB. The strands in NSB’s main cable are prefabricated parallel wire strands (PPWS). There are 87 strands in each main cable passing through two cable pylons from the Yibin anchorages to the Luzhou anchorages and two more strands are added to each main cable in the side spans. Each strand is composed of 127 high strength galvanized steel wires, of which the diameter and nominal tensile strength are 5.1 mm and 1670 MPa, respectively. Thus, the cross-section area of each main cable in the anchor span is 230,783 mm^2^ (89 × 2593 mm^2^). Figure 2a shows the saddle of the Yibin downstream anchorage. Each separate strand is anchored individually by using a single anchoring system as shown in Figure 2b.

The NSB, which was open to traffic in 2012, is located in the southern Sichuan Province in China. This bridge forms a vital transportation link over the upper Yangtze River from Yibin City to Luzhou city, see Figure 1. The bridge is a suspension bridge with steel box girders and the main span of 820 m. The 3-span arrangement of this bridge is 192 + 820 + 176 m [28].

### 2.2. Monitoring System of Cable Strands’ Tension Force

**Figure 2:**
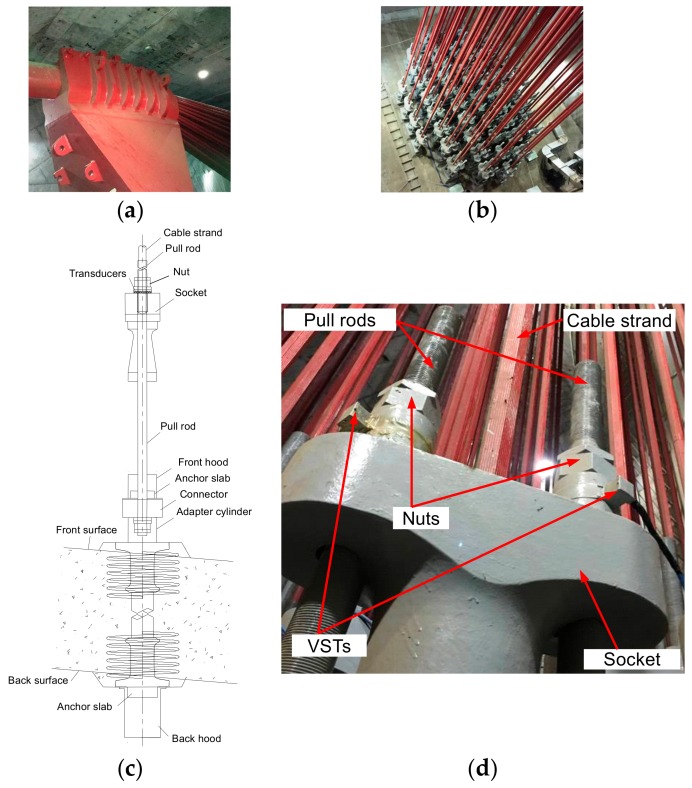
Anchoring system in the anchor span: (**a**) Saddle in Yibin downstream anchorage; (**b**) Front anchor surface in Yibin downstream anchorage; (**c**) Transducers’ locations and anchor system; (**d**) Two transducers to monitor one strand’s tension force.

In the main cable’s anchor span, the separation of cable strands provides opportunity to evaluate the main cable’s tension by monitoring several cable strands’ tension forces. As shown in Figure 2c,d, two VSTs were mounted between the socket and nuts of pull rods for a single cable strand. The transducers can measure the compression forces between the nuts and socket. Obviously, the strand’s tension force equals the sum of two transducers’ compression forces.

**Figure 3:**
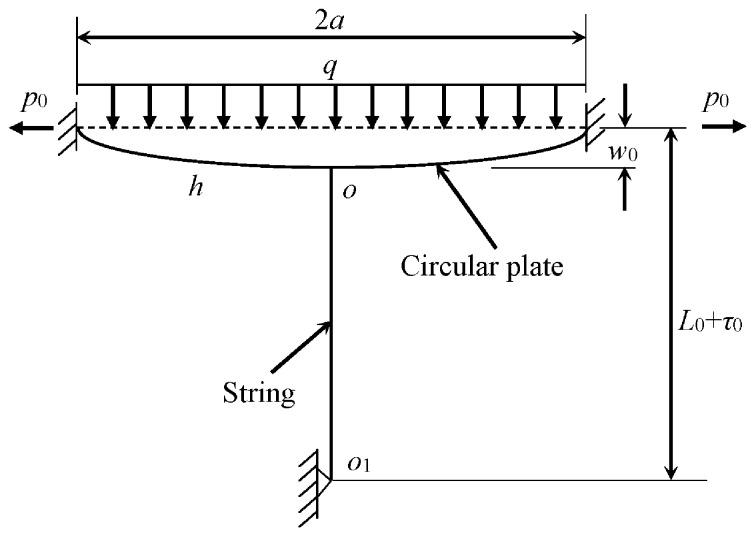
Schematic of the vibrating string transducer.

When a pressure is applied on a VST sensor, the deformation of the sensor causes variations of the tension forces of the strings inside the sensor, which lead to changes of the strings’ frequencies [23,24]. By measuring the frequencies of the stretched strings inside a VST sensor, the pressure can be computed through the pressure-frequency relationship. The key parts of vibrating string transducer consist of a thin circular plate (radius *a*, thickness *h*) and string, see Figure 3. One end of the string of length *L*_0_ is connected to the center of the plate *o*. The other end of the string is moved along its line in a displacement *τ*_0_ before being fixed to point *o*_1_. The in-plane uniform tension *p*_0_ is applied around the edge of the plate. The string, connected to the center of the plate, is pre-stretched. The transverse static pressure *q* applied on the top surface of the plate brings additional transverse displacement of the plate. The change of the central transverse displacement of the plate *w*_0_ results in variation of both the string’s tensile force and natural frequencies. The measured fundamental resonant frequency of the string will therefore indicate the static pressure applied on the plate [24].

**Figure 4:**
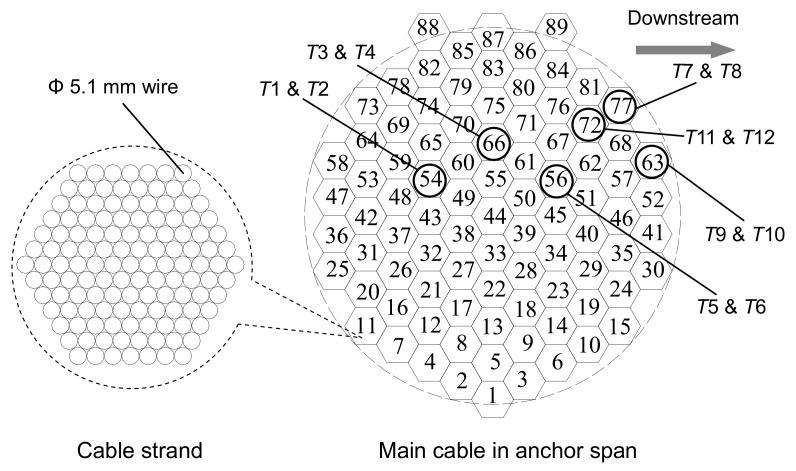
Strand arrangement and transducer placement in main cable of anchor span.

During the construction period, VSTs were employed to monitor the tension forces during the installation process of cable strands to make sure that the sum of two pull rods’ tension forces reaches the design value. After installing cable strands, some transducers have been kept continuing monitoring the cable tension during the service time. Installing VSTs to the separate strands is easy to implement. First, the calculated tension forces can help to determine the measuring range of the transducers. During construction period, the transducers should be placed to the pull rods shown in Figure 2d. Once the tension forces of the strands reach the design values, the nuts should be tightened immediately and then the transducers can’t be removed. Figure 4 shows six cable strands, on each of which are instrumented with two transducers.

### 2.3. Estimation of Main Cable’s Tension Force

As shown in Figure 2c,d, transducers are mounted between the socket and pull rods’ nuts and each transducer measures the change of the compression force between cable strand’s socket and pull rod’s nut. The SHMS of the NSB adopted 4-string transducers to monitor the tension force of the main cable. Figure 3 just shows a string to demonstrate the working mechanism of the VSTs. In practical engineering, usually a multi-string setup of the VSTs is designed to address the possible uneven loading. The transducers’ measuring range and sensitivity are 1000 kN and ±1 kN, respectively. According to the basic theory of vibrating string transducer for pressure monitoring [24], the tension force of the main cable can be estimated following the procedures summarized below.

For a single transducer, the current strain of the *j*th string can be calculated based on its fundamental resonant frequency:

(1) $$\varepsilon_{j}=K\times f_{j}^{2},\;j=1,2,3\;\mathrm{or}\;4$$

where *ε_j_*, of which the unit is *με*, is the strain of the *j*th string. The coefficient *K* of the strings is 0.00084107 for the type of transducer. *f_j_* is the fundamental resonant frequency of the *j*th string (Hz). The current average strain of 4 strings in a VST can be obtained by:

(2) $$\varepsilon =\frac{\varepsilon_{1}+\varepsilon_{2}+\varepsilon_{3}+\varepsilon_{4}}{4}$$

The change of strain Δ*ε* is:

(3) $$\Delta \varepsilon =\varepsilon -\varepsilon_{0}$$

where *ε*_0_ is the initial strain. The strain change after eliminating temperature effect is defined as:

(4) $$\Delta \varepsilon_{t}=\Delta \varepsilon +\left(t-t_{0}\right)\times \left(C_{t}-C_{x}\right),$$

where, Δ*ε_t_* is the strain change after eliminating temperature effect (με); *t* is the current temperature (°C); *t*_0_ is the baseline temperature (°C); Temperature-strain coefficient of transducer’s body *C_t_* is 11.7 με/°C; Temperature-strain coefficient of transducer’s string *C_x_* is 12.2 με/°C. The coefficients *K*, *C_t_*, and *C_x_*, which are physical characteristics of the transducer’s materials, are provided by the manufacture. The compression force *N* applied to the transducer has a linear relationship with the strain change Δ*ε_t_*.

(5) $$N=a+b\times \Delta \varepsilon_{t},$$

where *a* (kN) and *b* (kN/με) are the correction and calibration coefficients, respectively. Generally, each transducer has its own correction and calibration coefficients. Table 1 lists coefficients of all the transducers also provided by the manufacture.

The transducers record one data value per every 2 min. The tension force of main cable *T* can be approximately estimated as:

(6) $$T=89\times \frac{\sum T_{Si}}{6}=89\times \frac{\sum_{l=1}^{12}N_{l}}{6}\left(i=54,\;56,\;63,\;66,\;77,\;\mathrm{and}\;72;l=1,\;2,\ldots ,12\right),$$

(7) $$\begin{array}{c} T_{S54}=N_{1}+N_{2},\;T_{S66}=N_{3}+N_{4}\\ T_{S56}=N_{5}+N_{6},\;T_{S77}=N_{7}+N_{8}\\ T_{S63}=N_{9}+N_{10},\;T_{S72}=N_{11}+N_{12} \end{array}$$

where *T_Si_* represents the tension force of the *i*th cable strand (*I* = 54, 56, 63, 66, 77, and 72). *N_l_* (*l* = 1, 2, 3,…, 12) obtained by using Equation (5) represents the compression force measured by transducer *l*.

## 3. Evaluation Methods

### 3.1. Uniformity of Cable Strands’ Tension Forces

During the service period, great attention should be paid to the uniformity of cable stands’ tension forces in the anchor span. If there is significant non-uniformity among the cable strands’ tension forces, it means some strands experience more tension forces than what are expected to be. Thus, the performance of the main cable and even whole bridge will be adversely affected. Coefficient of variation (COV) is employed to assess the non-uniformity among the measured tension forces of multiple strands. At a given time, the COV could be calculated as:

(8) $$E_{T}=\frac{\sum T_{Si}}{6}\left(i=54,\;56,\;63,\;66,\;77,\;\mathrm{and}\;72\right),$$

(9) $$\sigma_{T}=\sqrt{\frac{\sum {\left(T_{Si}-E_{T}\right)}^{2}}{6}},$$

(10) $$\mathrm{COV}=\frac{\sigma_{T}}{E_{T}},$$

where *E_T_* and *σ_T_* are the mean value and standard deviation of the current tension forces of the selected strands; *T_si_*, which has been defined in Equation (7), is the tension force of strand *i*. It is obvious that the more COV indicate higher non-uniformity.

### 3.2. Thresholds of Main Cable’s Safety Factor

It is well known that the actual loads on the bridge during the service period are totally different from the design loads. The suspension bridge may encounter some extreme loads such as overloading trucks and strong winds. Besides, corrosion fatigue due to the adverse operation circumstances may result in cross section loss of main cable. These unfavorable factors will be harmful to main cable’s safety. Hence, the safety factor is often used to evaluate the current load carrying capacity of main cable. Base on Chinese standard JTG TD65-05-2015 [29], the safety factor of a main cable can’t be less than 2.5. This provision was determined from the previous studies and engineering experience [30]. The safety factor *K_s_* of a main cable is defined as:

(11) $$K_{S}=\frac{\sigma_{n}}{\sigma }=\frac{\sigma_{n}\times A}{T}\geq 2.5$$

where *σ_n_*, of which the value is 1670 MPa, is the nominal tensile strength of main cables’ steel wires. *σ* is the calculated stress of steel wires. The cross-sectional area of the main cable *A* is 230,783 mm^2^. *T* is the estimated tension force of main cable.

According to JTG D60-2004 [31], the design stress of a main cable is related to the dead load, vehicle load, pedestrian load, wind load and temperature effects. According to the bridge design documentation, the calculated tension force of the main cable in the Yibin anchor span is 134,157 kN. Thus, the calculated stress of steel wires *σ* is 581 MPa. By using Equation (11) the design safety factor of the main cable is 2.87. Hence, two thresholds of the safety factor are established in this study: the first level of safety factor is 2.87 and the second is 2.5. When the actual safety factor is lower than the first level, it suggests that the operational loads are more than the design loads. When lower than the second level, it suggests that the design code requirement is not met and serious attention should be paid to the possible performance degradation and safety of main cable.

## 4. Analysis of Monitoring Data

### 4.1. One-Day Monitoring Data

The data measured on 1 December 2015 is selected and processed in this section. Strand *S*56 is taken as the study object to demonstrate the estimation process of main cable’s tension force from the original monitoring data. A temperature sensor is also integrated into each transducer to measure the temperature data. The transducers are specifically designed to measure and output the real-time data including strings’ frequencies, temperature, and compression force. Table 2 lists strings’ frequencies and baseline temperatures of Transducer *T*5 (attached to *S*56), which were obtained through product calibration by the manufacturer before delivery. According to Equations (1) and (2), the beginning strain of *T*5 can be obtained.

**Figure 5:**
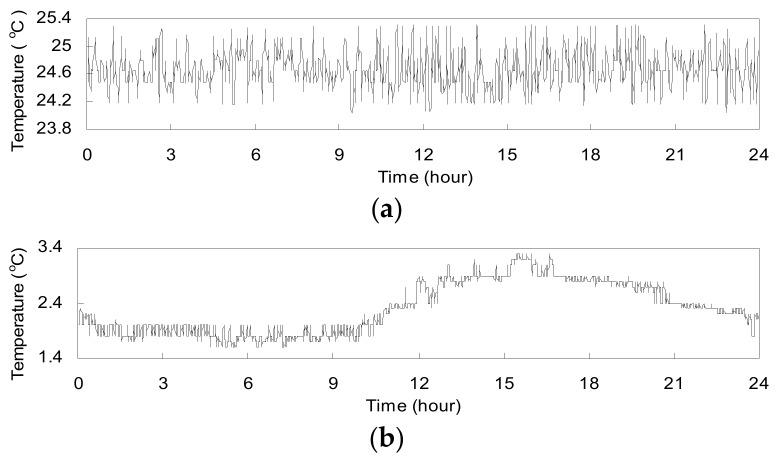
Temperature measurements: (**a**) Temperature measured by transducer *T*5 attached to cable strand *S*56; (**b**) Environmental temperature.

Figure 5a shows the actual temperature measurements by *T*5 in the bridge service stage. Figure 5b gives the environmental temperature measured by a sensor installed at the middle of the NSB’s deck. There is apparent difference between the environmental temperature and the temperature measured by *T*5. The temperature in Figure 5b shows a typical day-and-night change of air temperature. However, there is little daily variation of the temperature in Figure 5a and it seems that there is no influence of the environmental temperature on the measured temperature by *T*5. The reason is that the ground anchorage, isolated from the outside environment, is in a relatively closed space.

**Figure 6:**
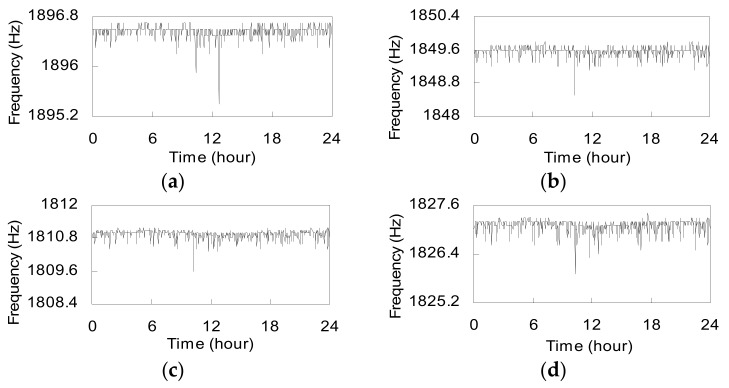
Fundamental resonant frequencies of strings in transducer *T*5: (**a**) string 1; (**b**) string 2; (**c**) string 3; (**d**) string 4.

**Figure 7:**
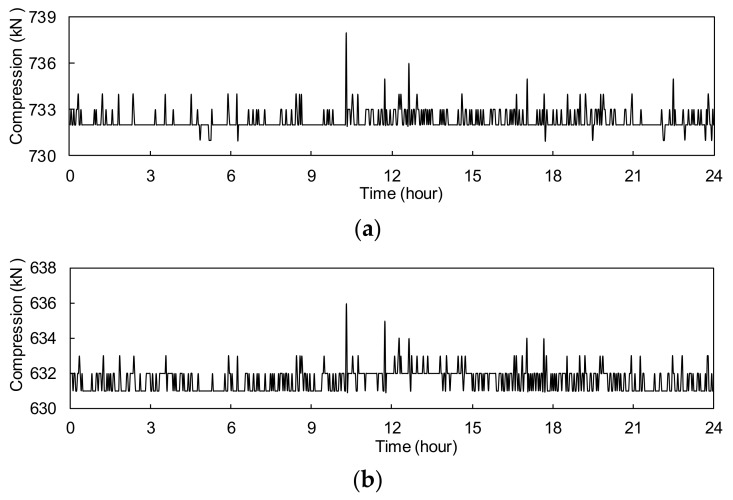
Compression force: (**a**) *T*5; (**b**) *T*6.

Figure 6 gives the measured frequencies of strings in *T*5. It is found that the measured frequencies in Figure 6 are lower than those listed in Table 2. The lower frequencies suggest that there is additional pressure imposed on the transducer [23,24]. The strings in transducers were pre-stretched and the pressure causes a reduction in string tension. Figure 7 shows the transducers’ compression forces calculated by using Equations (4) and (5). The compression forces of *T*5 and *T*6 vary in small ranges: from 731 kN to 738 kN and 631 kN to 636 kN, respectively.

**Figure 8:**
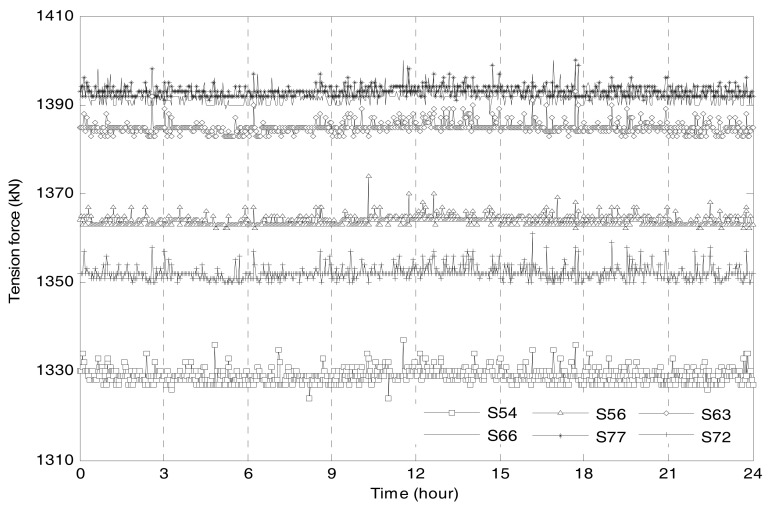
Tension forces of cable strands on 1 December 2015.

The tension force of *S*56, obtained based on compression forces of *T*5 and *T*6, is shown in Figure 8. The results of the other strands are also shown in the figure. Table 3 lists statistical characteristics of the selected strands’ tension forces measured on 1 December 2015. The data reveals that the largest tension forces were observed in strand *S*66 and *S*77 while Strand *S*54 experienced the least tension force. For each single strand, the daily variation of tension force is less than 15 kN. It can be seen in Table 3 that the maximum and minimum mean values are 1393 kN for S77 and 1329 kN for S54, respectively. The differences among the strands’ tension forces are very small.

**Figure 9:**
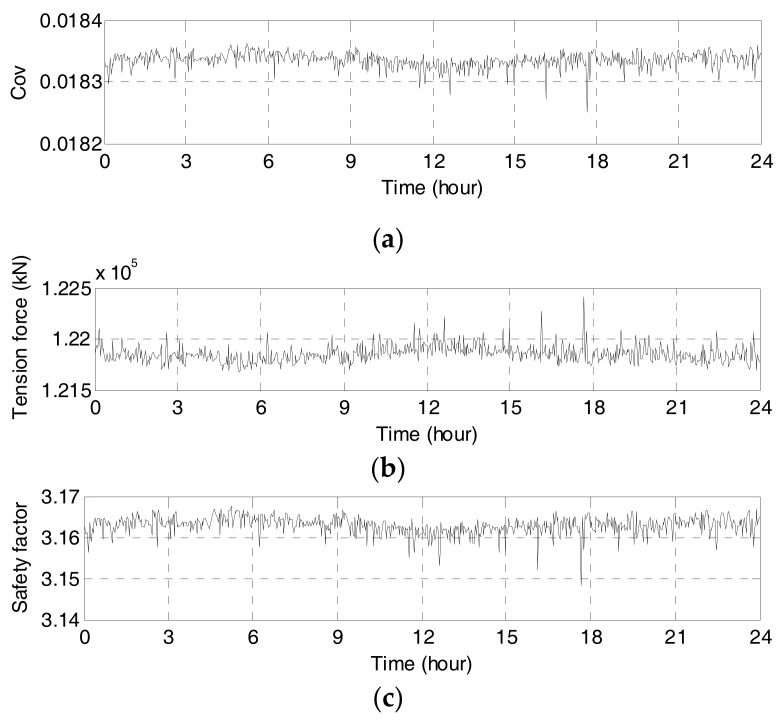
Assessment results on 1 December 2015: (**a**) COV of cable strands’ tension forces; (**b**) Tension forces of main cable in anchor span; (**c**) Safety factor of main cable.

For every 2 min, Equation (10) is used to calculate a COV of the six cable strands’ tension forces. The COV curve of the tension force is plotted in Figure 9a. The COV of 1 December 2015 changed in the range of 1.82% to 1.84%. The results indicate that the tension force uniformity of the instrumented cable strand is well maintained according to the analysis of 1-day short-term monitoring data.

The main cable’s tension forces of 1 December 2015 are estimated by using Equation (6) per every two minutes. As shown in Figure 9b the maximum and minimum tension forces are 122,420 kN and 121,680 kN, respectively. The safety factors are calculated by using Equation (11). The safety factors of 1 December 2015 in Figure 9c vary in the range between 3.15 and 3.17. Since the safety factors are higher than the first level of safety factor criterion, the monitored tension force of the main cable is within the designed value, exhibiting acceptable performance.

### 4.2. Long-Term Monitoring Data

**Figure 10:**
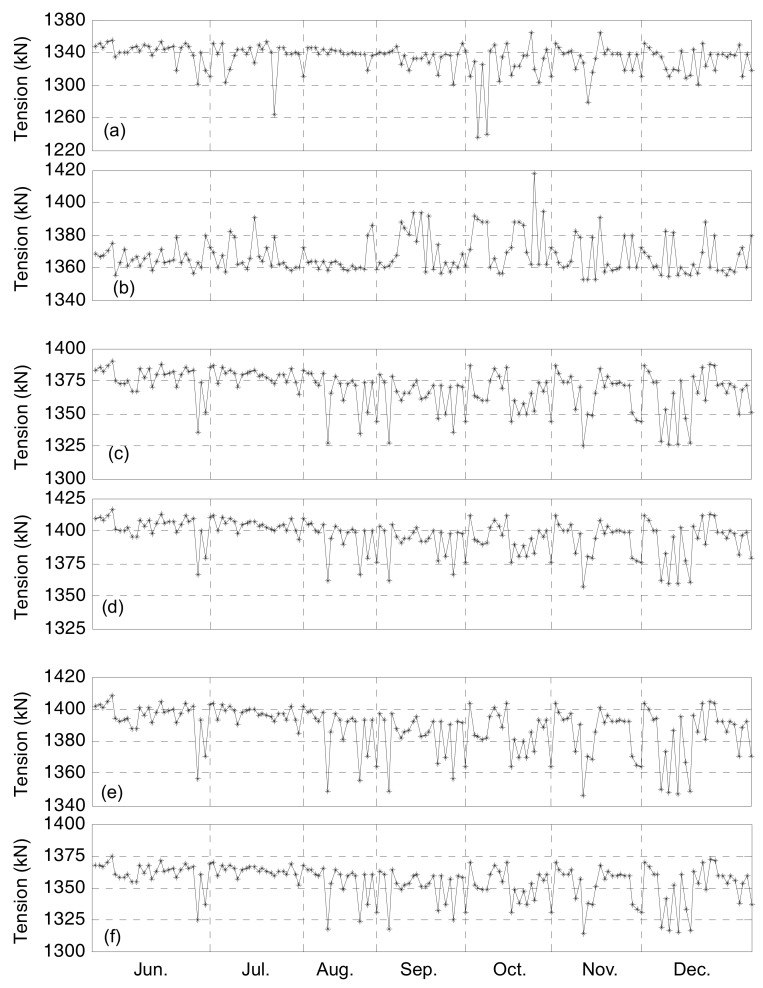
Daily averaged tension forces: (**a**) S54; (**b**) S66; (**c**) S56; (**d**) S77; (**e**) S63; (**f**) S72.

After the NSB was open to traffic, the VST-based system began to monitor the anchor strands’ tension forces. In this study, a long-term monitoring data from June to December of 2015 is selected. Due to power and communication problems, there was some data loss on several days during the 7-month period. Totally there are data for 162 days which are complete, including 29, 23, 18, 22, 21, 22 and 27 days in June through December of 2015, respectively. For each day, the data of every cable strand’s tension force are averaged to obtain a mean value and there are 162 data points in each curve shown in Figure 10.

**Figure 11:**
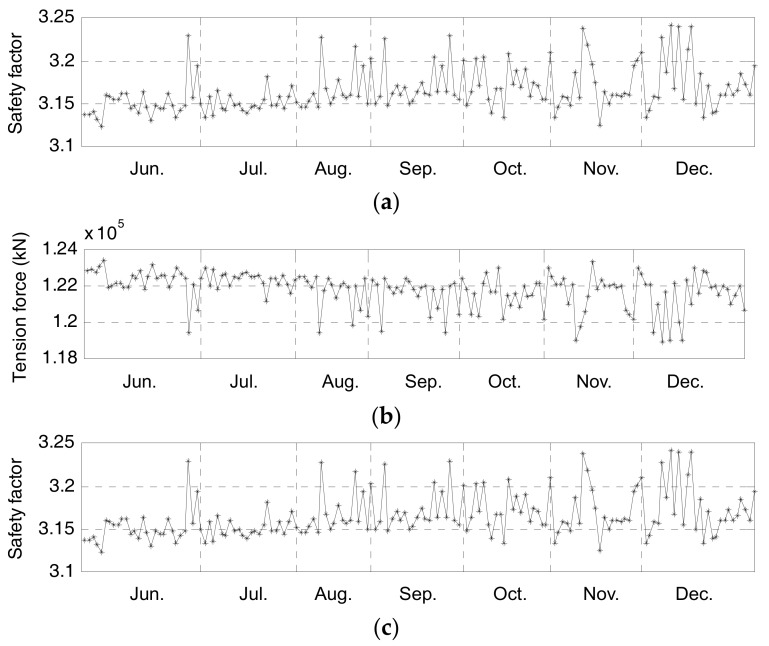
Long-term assessment results: (**a**) Daily COV of instrumented cable strands’ tension forces; (**b**) Daily averaged tension forces of main cable in anchor span; (**c**) Daily safety factor of main cable.

It can be observed that the tension forces throughout the 7-month period vary randomly and the statistical characteristics of the data in Figure 10 are listed in Table 4. The largest tension forces are observed on *S*66 and *S*77 and the least tension force appears on *S*54. The variation of *S*54’s tension force, of which the value is 127.9 kN, is the largest. On the other hand, the variations of the tension forces for the rest of the strands are about 60 kN. The daily COVs throughout the 7 months, varying between 1.25% and 1.35%, are shown in Figure 11a. With small COVs, the uniformity of the monitored tension forces of the instrumented cable strands is deemed excellent based on the long-term monitoring data.

Figure 11b gives the daily averaged tension forces of the main cable, which are calculated following Equation (6) based on the tension forces shown in Figure 10. The mean, maximum and minimum tension forces are 121,750 kN, 123,380 kN and 118,880 kN, respectively. Hence, the variation of the main cable’s tension force, of which the value is 4500 kN, is only 3.7% of the mean tension force during the seven months. Such phenomenon is consistent with the existing knowledge about suspension bridges that most of the main cable force comes from permanent loads (e.g., bridge dead loads) and only a small portion is caused by the action from other loads [30,32].

Finally, the safety factor curve during the seven months is plotted in Figure 11c. The maximum and minimum safety factors are 3.24 and 3.12, respectively. Obviously, the minimum safety factor is not only larger than 2.5—the second level of safety factor from the design specification, but also larger than 2.87—the first level of safety factor related to the design actions. The results indicate that the original design, which was carried out based on the design loads or actions, ensures enough capacity of the NSB’s main cables during the bridge service period. Because the NSB could still be treated as a new-built suspension bridge, there is no abnormal data in the long-term monitoring of this study. Further long-term monitoring over years can be expected to disclose more information after deterioration occurs.

## 5. Conclusions

A health monitoring and performance assessment strategy for the main cable force of a suspension bridge was proposed. The proposed technology monitors tensions of a series of strands in the anchor span rather than the entire main cable, which is acceptable for in-service monitoring of the main cable’s tension.

Conventional VSTs were adopted to monitor the main cable’s tension force for a suspension bridge in this study. Such transducers have many advantages, such as low cost, reliable technology and good accuracy. Low-cost monitoring systems based on vibrating string-sensing technology have shown their reliable performance and potential in long-term monitoring of main cables’ tension.

With the monitoring data, the performance evaluation of main cable can be carried out by focusing on both overall cable force and uniformity of forces among different strings. The thresholds of the main cable’s safety factor were established.

The proposed technology was demonstrated on a prototype long-span suspension bridge and the one-day and seven-month monitoring data were analyzed. The results show that the proposed methods can provide an approximate estimation of main cables’ tension forces. For the prototype suspension bridge, when more transducers are mounted to the strands, more accurate estimation of the main cable’s tension force can be obtained. The lesson from this study is that when more data is collected, more information about the in-service performance of the main cable can be revealed, and the conventional sensing technique can still play an effective role in health monitoring for main cables of suspension bridges.

## Figures and Tables

**Table 1:** Correction and calibration coefficients.

Strand NO.	54		66		56		77		63		72	
VST NO.	1	2	3	4	5	6	7	8	9	10	11	12
*a*	−12.7	−42	−9.1	−15	−7.3	−9.7	−6	−9.8	−6.13	−12.3	−8.6	−12
*b*	1.595	1.591	1.569	1.538	1.584	1.540	1.548	1.536	1.552	1.553	1.552	1.587

**Table 2:** Initial frequencies and baseline temperatures.

*f*_1_	*f*_2_	*f*_3_	*f*_4_	Baseline Temperature *T*_0_	Initial Strain
1989 Hz	1988 Hz	1990 Hz	1991 Hz	33 °C	3329.0 με

**Table 3:** Statistical characteristics of tension forces on 1 December 2015.

Cable Strand	*S*54	*S*66	*S*56	*S*77	*S*63	*S*72
Min value (kN)	1324	1388	1362	1391	1383	1349
Max value (kN)	1337	1400	1374	1400	1393	1361
Mean value (kN)	1329	1392	1364	1393	1385	1352
Change range (kN) (Max − Min)	13	12	12	9	10	12

**Table 4:** Statistical characteristics of tension forces in 7 months of 2015.

Cable Strand	*S*54	*S*66	*S*56	*S*77	*S*63	*S*72
Min value (kN)	1236.7	1352.7	1324.8	1357.5	1346.4	1314.0
Max value (kN)	1364.6	1417.9	1390.4	1416.3	1407.8	1374.4
Mean value (kN)	1333.2	1367.5	1368.6	1396.2	1388.2	1354.5
Change range (kN) (Max − Min)	127.9	65.3	65.7	58.7	61.4	60.3
